# Role of toll-like receptor 4 in skeletal muscle damage in chronic limb-threatening ischemia

**DOI:** 10.1016/j.jvssci.2024.100194

**Published:** 2024-02-11

**Authors:** Ali Navi, Hemanshu Patel, Xu Shiwen, Daryll Baker, David Abraham, Janice Tsui

**Affiliations:** a Division of Surgery & Interventional Science, University College London, London, United Kingdom; b Centre for Rheumatology & Connective Tissue Disease, University College London, London, United Kingdom

**Keywords:** Critical limb-threatening ischemia (CLTI), Inflammation, Peripheral arterial disease (PAD), Skeletal muscle, Toll-like receptor (TLR)

## Abstract

**Objective:**

Toll-like receptors (TLRs) are key pattern recognition receptors in the innate immune system. In particular, the TLR4-mediated immune response has been implicated in ischemia-induced tissue injury. Mounting evidence supports a detrimental role of the innate immune system in the pathophysiology of skeletal muscle damage in patients with chronic limb-threatening ischemia (CLTI), in whom patient-oriented functional outcomes are poor. The overall aim of this study was to investigate the potential role of TLR4 in skeletal muscle dysfunction and damage in CLTI.

**Methods:**

The role of TLR4 in ischemic muscle was investigated by (1) studying TLR4 expression and distribution in human gastrocnemius muscle biopsies, (2) evaluating the functional consequences of TLR4 inhibition in myotubes derived from human muscle biopsies, and (3) assessing the therapeutic potential of modulating TLR4 signaling in ischemic muscle in a mouse hindlimb ischemia model.

**Results:**

TLR4 was found to be expressed in human muscle biopsies, with significant upregulation in samples from patients with CLTI. In vitro studies using cultured human myotubes demonstrated upregulation of TLR4 in ischemia, with activation of the downstream signaling pathway. Inhibition of TLR4 before ischemia was associated with reduced ischemia-induced apoptosis. Upregulation of TLR4 also occurred in ischemia in vivo and TLR4 inhibition was associated with decreased inflammatory cell infiltration and diminished apoptosis in the ischemic limb.

**Conclusions:**

TLR4 is upregulated and activated in ischemic skeletal muscle in patients with CLTI. Modulating TLR4 signaling in vitro and in vivo was associated with attenuation of ischemia-induced skeletal muscle damage. This strategy could be explored further for potential clinical application.

**Article Highlights**

• **Type of Research:** Human tissue analyses, in vitro and in vivo models
• **Key Findings:** Pharmaceutical inhibition of the Toll-like receptor 4 pathway might be of clinical significance in patients with peripheral arterial disease, where it could be used as an adjunctive treatment to improve outcomes of treatments at different stages of the disease.
• **Take Home Message:** Poor functional outcomes in patients with chronic limb-threatening ischemia, even after successful revascularization, have highlighted the importance of other contributory factors involved in the pathophysiology of peripheral arterial disease.

The prevalence of peripheral arterial disease (PAD) is growing and is a global health care burden with important public health implications.^1^^,^^2^ Although ≤38% of these patients are asymptomatic, the rest experience some degree of symptoms ranging from mild intermittent claudication to the most severe form that is chronic limb-threatening ischemia (CLTI). Successful revascularization in patients with CLTI contributes to limb salvage; however, this process does not equate to a return to premorbid ambulatory or occupational status.^3^ This finding suggests that adjunctive treatments in addition to revascularization to improve both clinical and patient-focused outcomes should be explored.

It has been demonstrated that changes within the lower limb skeletal muscle occur in patients with PAD and contribute to the clinical manifestations and poor functional outcomes.^4^, ^5^, ^6^ Skeletal muscle forms the bulk of the lower limb and plays a key part in its function. Further, skeletal muscle has a role as a paracrine signaling organ, whereby pro-inflammatory cytokines released from the damaged muscle can trigger a systemic inflammatory response.

The innate immune system recognizes damage-associated molecular patterns, upregulated in any tissue damage, via pattern recognition receptors. Among the better-characterized pattern recognition receptors are the Toll-like receptors (TLRs). The immune response mediated by TLRs is protective in most cases. However, if inflammation becomes chronic, excessive tissue damage may occur, contributing to the pathogenesis of diseases such as atherosclerosis.^7^^,^^8^ Recognition of damage-associated molecular patterns by TLRs results in the recruitment of adaptor proteins and activation of downstream signaling pathways: the MyD88-dependent and TRIF-dependent pathways. Each pathway starts the activation of specific transcription factors such as nuclear factor κB (NF-κB), Jun N-terminal kinase (JNK), and mitogen-activated protein kinases, which are required for inflammatory gene transcription. This in turn results in the release of a wide range of cytokines and inflammatory markers such as IL1, IL6, IL8, tumor necrosis factor-α (TNF-α), interferon-α and interferon-β.^9^

TLR4 has a pathogen detection role and acts as a monitoring receptor in the detection of tissue injury. The release of TLR4 endogenous ligands is associated with TLR4 activation and the corresponding inflammatory response can lead to excessive tissue damage. Exogenous inhibition of TLR4 in an animal model of myocardial ischemia has been associated with decreased ischemic injury and inflammation.^10^

The overall aim of this study was to investigate the potential role of TLR4 in skeletal muscle dysfunction in CLTI. The role of TLR4 in ischemic muscle was studied with three aims: (1) to explore TLR4 expression and distribution in muscle biopsies from patients with CLTI, (2) to investigate the TLR4 pathway and functional consequences of TLR4 inhibition in myotubes derived from human muscle biopsies, and (3) to evaluate the therapeutic potential of modulating TLR4 signaling in ischemic muscle using a relevant mousehind limb ischemia model.

## Methods

Gastrocnemius biopsies were taken from the medial head of the gastrocnemius muscle from patients undergoing major lower limb amputation for CLTI (ischemic group) and from patients with no PAD undergoing saphenous vein harvesting for coronary artery bypass surgery (control group). Western blot analysis and immunostaining were carried out to study TLR4 expression and distribution, and to explore the downstream signaling pathways.

Further, human myoblasts were isolated, cultured to myotubes, and then exposed to simulated ischemia. The isolated myoblasts were examined every 48 hours to monitor for 80% confluence before induction to differentiate into myotubes. Enzyme-linked immunosorbent assay (ELISA), Western blot, and immunostaining were used to study the role of TLR4 signaling pathway inhibition in ischemia-induced apoptosis and cytokine release from myotubes. To study the therapeutic potential of modulating TLR4 signaling in ischemic skeletal muscle, a mouse model of hindlimb ischemia was used. The femoral artery was ligated with a sterile 7-0 Prolene suture at the level of the inguinal ligament proximally and just above the popliteal artery distally. All the side branches were ligated with 7-0 Prolene suture. Laser Doppler and immunostaining were carried out to assess the effect of TLR4 inhibition on skeletal muscle damage in vivo. For in vivo studies, animals were sacrificed (n = 6 per ischemic groups and n = 3 per sham groups at each time point). This number was chosen based on published studies of TLR changes in ischemia as well as studies employing similar analyses in mouse hindlimb ischemia models.^10^^,^^11^ JMP 11.0.0 software (SAS Institute, Cary, NC) was used to present, describe, and analyze the data from this study. Further information on the methods and materials in this study is detailed in the Supplemental Materials.

## Results

### Human tissue experiments

**Fig 1:**
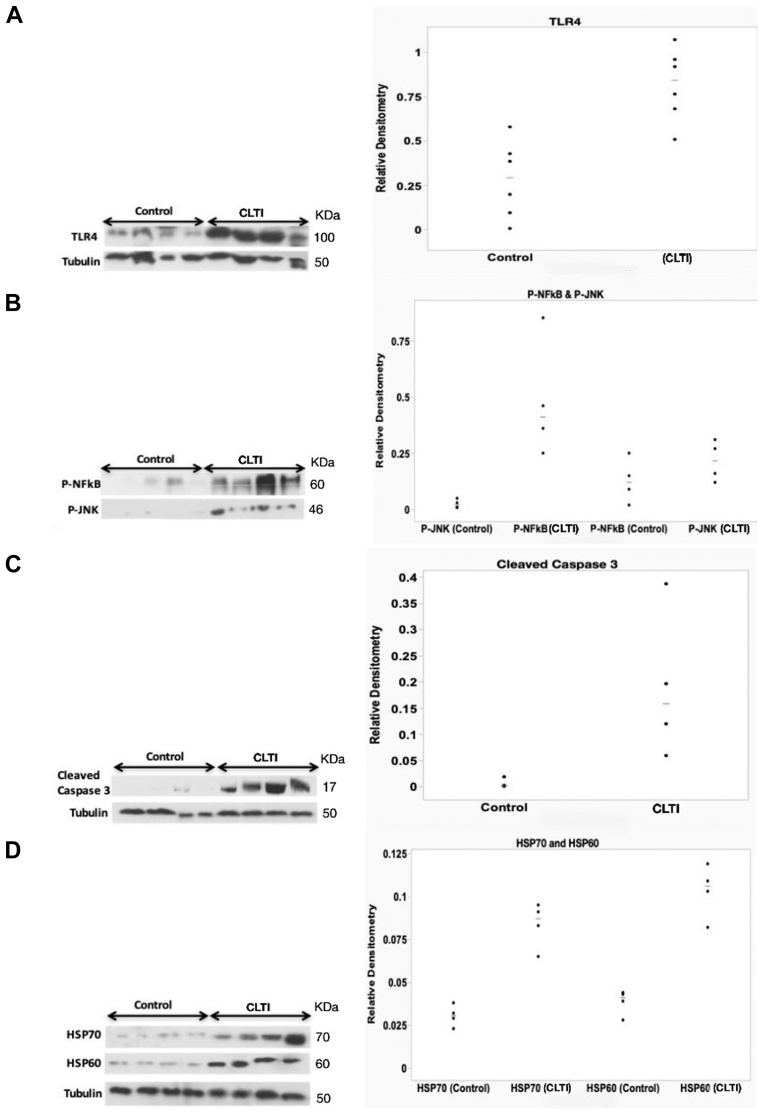
(**A**) Representative immunoblots from biopsied muscles for Toll-like receptor 4 (*TLR4*) and tubulin loading control and densitometric analysis of the TLR4 immunoblots (n = 6; *P* < .05; Mann-Whitney *U* test). (**B**) Representative immunoblots for phosphorylated nuclear factor κB (*P-NFκB*) and Jun N-terminal kinase (*P-JNK*) and densitometric analysis of the P-NFκB and P-JNK immunoblots (n = 4; *P* < .05; Mann-Whitney *U* test, between control and chronic limb-threatening ischemia [*CLTI*] samples). (**C**) Representative immunoblots from muscle biopsies for cleaved caspase 3 and tubulin loading control and densitometric analysis of the cleaved caspase 3 immunoblots (n = 4; *P* < .05; Mann-Whitney *U* test). (**D**) Representative immunoblots from muscle biopsies for HSP70, HSP60, and tubulin loading control and densitometric analysis of the HSP70 and HSP60 immunoblots (n = 4; *P* < .05; Mann-Whitney *U* test; between control and CLTI samples).

The patient groups differed only in terms of the presence of PAD symptoms and reduced ankle-brachial pressure index (Table I). Immunofluorescence staining demonstrated that TLR4 is expressed on endothelium (CD31), neutrophils (CD43), and macrophages (CD68) in muscle biopsies from patients with CLTI. Western blot analyses of muscle homogenates showed upregulation of TLR4 (median, 0.84; range, 0.50-1.07) in ischemic samples vs nonischemic samples (median, 0.29; range, 0.007-0.57; n = 6; *P* < .05; Mann-Whitney *U* test) (Fig 1). Transcription factors in human muscle biopsies were quantified showing increased phosphorylated NF-κB (median, 0.41; range, 0.25-0.85) and phosphorylated JNK (pJNK) (median, 0.21; range, 0.12-0.31) in ischemic samples vs nonischemic samples (pNF-κB, median, 0.12; range ,0.02-0.25; pJNK, median, 0.02; range, 0.009-0.05, n = 4; *P* < .05; Mann-Whitney *U* test) (Fig 1). Western blot analyses of cleaved caspase 3 expression were performed to measure apoptosis within the muscle and this was found to be increased in ischemic samples (median, 0.15; range, 0.059-0.387) vs nonischemic samples (median, 0.001; range, 0.0007-0.0187, n = 4; *P* < .05; Mann-Whitney *U* test) (Fig 1). Further, protein levels of heat shock protein (HSP)60 and HSP70, potential endogenous ligands of TLR4, were found to be elevated in CLTI biopsies (HSP60, median, 0.106; range, 0.082-0.119; HSP70, median, 0.087; range, 0.065-0.095) vs nonischemic biopsies (HSP60, median, 0.041; range,0.028-0.044; HSP70, median, 0.0305; range, 0.023-0.038, n = 4; *P* < .05; Mann-Whitney *U* test) (Fig 1).

**Table I:** Demographics of the two patient groups

Parameters	CLTI group (n = 6)	Control group (n = 6)	*P* value
Sex (male)	4	3	>.05
Age, mean, years	61.5	64.5	>.05
HTN	3	4	>.05
Diabetes	4	4	>.05
Smoking	3	2	>.05
eGFR (mean, mL/min/1.73 m^2^)	61	71	>.05
Hyperlipidemia	5	4	>.05
Symptoms of PAD	6	0	**<.05**
ABPI <0.9	6	0	**<.05**
CAD	4	6	>.05
Disease pattern	Infra inguinal	N/A	N/A

*ABPI*, ankle-brachial pressure index; *CAD*, coronary artery disease; *CLTI*, chronic limb-threatening ischemia; *eGFR*, estimated glomerular filtration rate; *HTN*, hypertension; *N/A*, not applicable; *PAD*, peripheral arterial disease.

Values are numbers unless otherwise noted. Boldface entries indicate statistical significance.

The two groups differed only in terms of the presence of PAD symptoms and reduced ABPI, where *P* < .05, Chi-square test

### Myotubes derived from patients without PAD

Western blot analyses of lysates of myotubes derived from patients with no PAD showed that TLR4 (median, 44; range, 26-53) and cleaved caspase-3 (median, 27; range, 18-33) were upregulated after exposure to simulated ischemia (*P* < .05; Mann-Whitney *U* test) compared with nonischemic myotubes from the same patient group (TLR4, median, 14; range, 6-18; cleaved caspase-3, median, 6; range, 4-8). ELISA assessment of the supernatants of myotubes derived from patients with no PAD, showed increased expression of IL-6 (median, 178 pg/mL; range, 133-269 pg/mL) and TNF-α (median, 199 pg/mL; range, 174-295 pg/mL) after simulated ischemia (*P* < .05; Mann-Whitney *U* test). Further, ELISA quantification of the supernatants of myotubes from patients with no PAD showed significant increase in both HSP60 and HSP70 (HSP60, median, 1240; range, 950-2200 vs median, 100; range, 50-1000; HSP70, median, 5900; range, 4500-7000 vs median, 1900; range, 800-3000), when exposed to simulated ischemia (*P* < .05; Mann-Whitney *U* test).

### Myotubes derived from patients with CLTI

**Fig 2:**
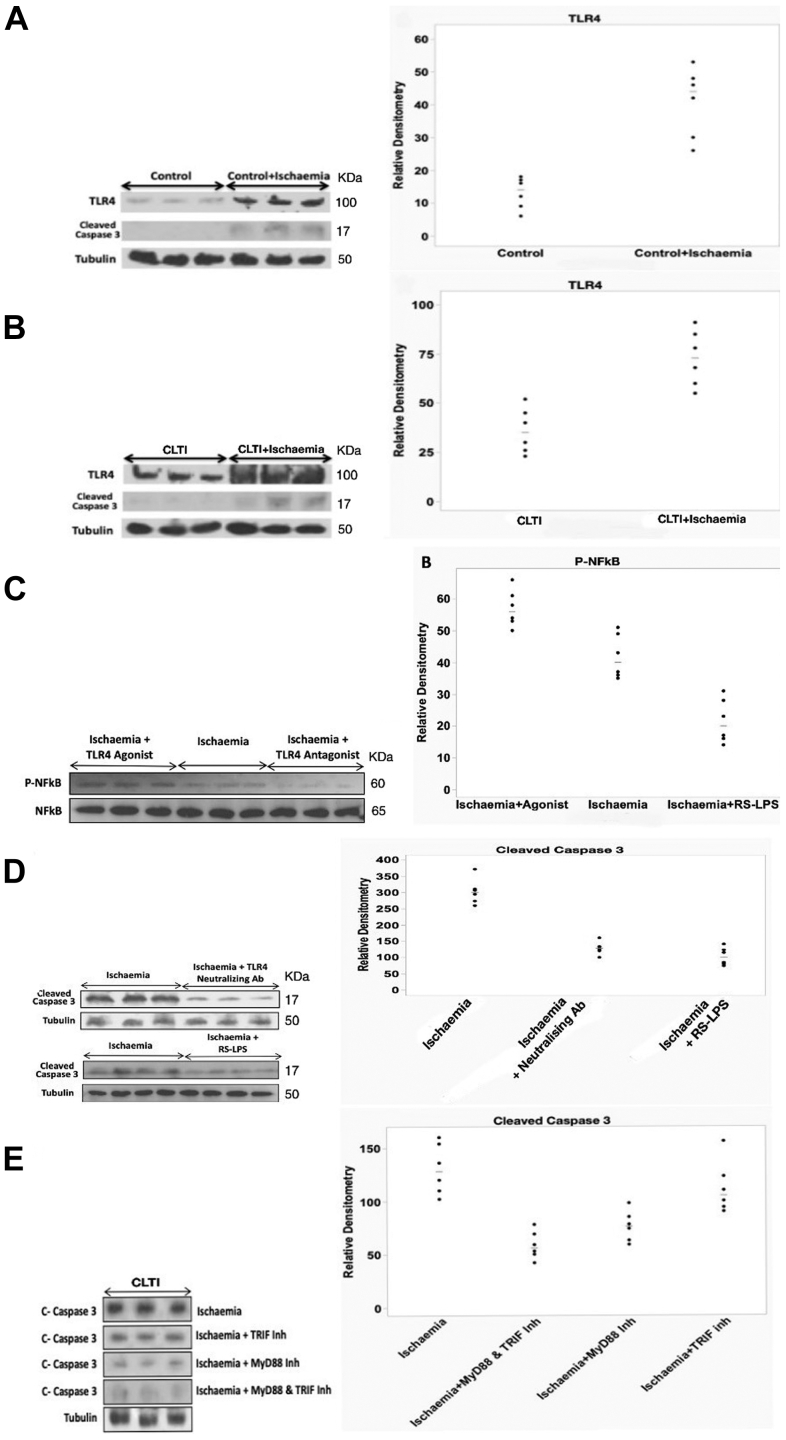
(**A**) Representative immunoblots from cultured myotubes from patients with no peripheral arterial disease (PAD) for TL4, cleaved caspase 3, and tubulin loading control and densitometric analysis of the TLR4 immunoblots (n = 4; *P* < .05; Mann-Whitney *U* test) (**B**) Representative immunoblots from cultured myotubes from patients with chronic limb-threatening ischemia (*CLTI*) for TL4, cleaved caspase-3, and tubulin loading control and densitometric analysis of the TLR4 immunoblots (n = 6; *P* < .05; Mann-Whitney *U* test). (**C**) Representative Western blots for phosphorylated nuclear factor κB (*P-NFκB*) with TLR4 agonist (synthetic LPS) or TLR4 antagonist (lipopolysaccharide from *Rhodobacter sphaeroides* [RS-LPS]) pretreatment before simulated ischemia in myotubes from patients with CLTI (**B**) Densitometric analyses of Western blots of P-NFκB with TLR4 agonist (synthetic LPS) or TLR4 antagonist (RS-LPS) pretreatment before simulated ischemia in myotubes from patients with CLTI (n = 6; *P* < .05; Mann-Whitney *U* test) (**D**) Representative Western blot of cleaved caspase-3 from patients with CLTI, which were pretreated with TLR4 antagonist (RS-LPS) or TLR4 neutralizing antibody before simulated ischemia (n = 6; *P* < .05; Mann-Whitney *U* test). (**E**) Effect of ischemia on myotubes from patients with CLTI. Representative Western blots of cleaved caspase-3 after inhibition of adaptor proteins and densitometric analyses of Western blots showing downregulation of cleaved caspase-3 after inhibition of MyD88 (n = 6; *P* < .05; Mann-Whitney *U* test).

The cultured myotubes from patients with CLTI showed similar findings in terms of up-regulation of TLR4 (median, 73; range 55-91 vs median, 35; range, 23-52; *P* < .05; Mann-Whitney *U* test) and cleaved caspase-3 (median, 28; range 21-47 vs median, 8.5; range, 5-13; *P* < .05; Mann-Whitney *U* test), and increased expression of IL-6 and TNF-α after simulated ischemia (IL-6; median, 207; range, 180-225 vs median, 35; range, 20-49; TNF-α, median, 393; range, 330-425 vs median, 14; range, 8-22; *P* < .05; Mann-Whitney *U* test) compared with nonischemic myotubes from the same patient group. Western blot analyses of the cell lysates confirmed the presence of both HSP60 and HSP70, and demonstrated up-regulation of these endogenous ligands in patients with CLTI, when exposed to simulated ischemia (HSP60, median, 15; range, 13-16 vs median, 5; range, 2.5-7; HSP70, median, 19; range, 17-21 vs median, 11; range, 7-14) (*P* < .05; Mann-Whitney *U* test). ELISA quantification of the supernatants showed a significant increase in both HSP60 and HSP70 in patients with CLTI (HSP60, median, 3800; range, 2700-4500 vs median, 1500; range, 700-2400; HSP70, median, 7000; range, 5700-8000 vs median, 3200; range, 2500-4800), when exposed to simulated ischemia (*P* < .05; Mann-Whitney *U* test). Myotubes from patients with CLTI expressed greater levels of TLR4 as compared with those from patients with no PAD after exposure to the simulated ischemia (Fig 2).

### TLR4 inhibition in myotubes derived from patients with CLTI

Pretreatment of myotubes from patients with CLTI using TLR4 antagonist before simulated ischemia reduced the expression of phosphorylated -NF-κB (P-NF-κB) (median, 20 pg/mL; range, 14-31 pg/mL) in ischemic cell lysates as compared with ischemic cell lysates with no pretreatment (median, 40 pg/mL; range, 35-51 pg/mL) (*P* < .05; Mann-Whitney *U* test) (Fig 2). Pretreatment of myotubes from patients with CLTI using TLR4 neutralizing antibody or TLR4 antagonist (lipopolysaccharide from *Rhodobacter sphaeroides*) before simulated ischemia reduced the expression of cleaved caspase 3 (TLR4 neutralizing antibody, median, 100; range, 74-141; TLR4 antagonist lipopolysaccharide from *Rhodobacter sphaeroides*, median, 128; range, 100-160) in ischemic cell lysates as compared with ischemic cell lysates with no pretreatment (median, 302; range, 259-371) (*P* < .05; Mann-Whitney *U* test) (Fig 2). Further, pretreatment with TLR4 agonist (LPS) was associated with increased expression of P-NF-κB (median, 56; range, 50-66 vs median, 40; range, 35-51) (*P* < .05; Mann-Whitney *U* test). The amount of P-NF-κB, the activated form of NF-κB, was normalized as a ratio to the total levels of NF-κB. ELISA quantification of the supernatants showed a significant decrease in expression of endogenous ligands HSP60 (median, 2230; range, 1400-2900 vs median, 3700; range, 2100-4500) and HSP70 (median, 3070; range, 2500-4250 vs median, 7000; range, 6250-8100), as well as inflammatory markers IL6 (median220; range, 200-255 vs median, 105; range, 70-155) and TNF-α (median, 398; range, 310-476 vs median, 145; range, 110-245), when the cells are pretreated with TLR4 antagonist before simulated ischemia (*P* < .05; Mann-Whitney *U* test) (Fig 2).

### Inhibition of adaptor proteins in myotubes derived from patients with CLTI

**Fig 3:**
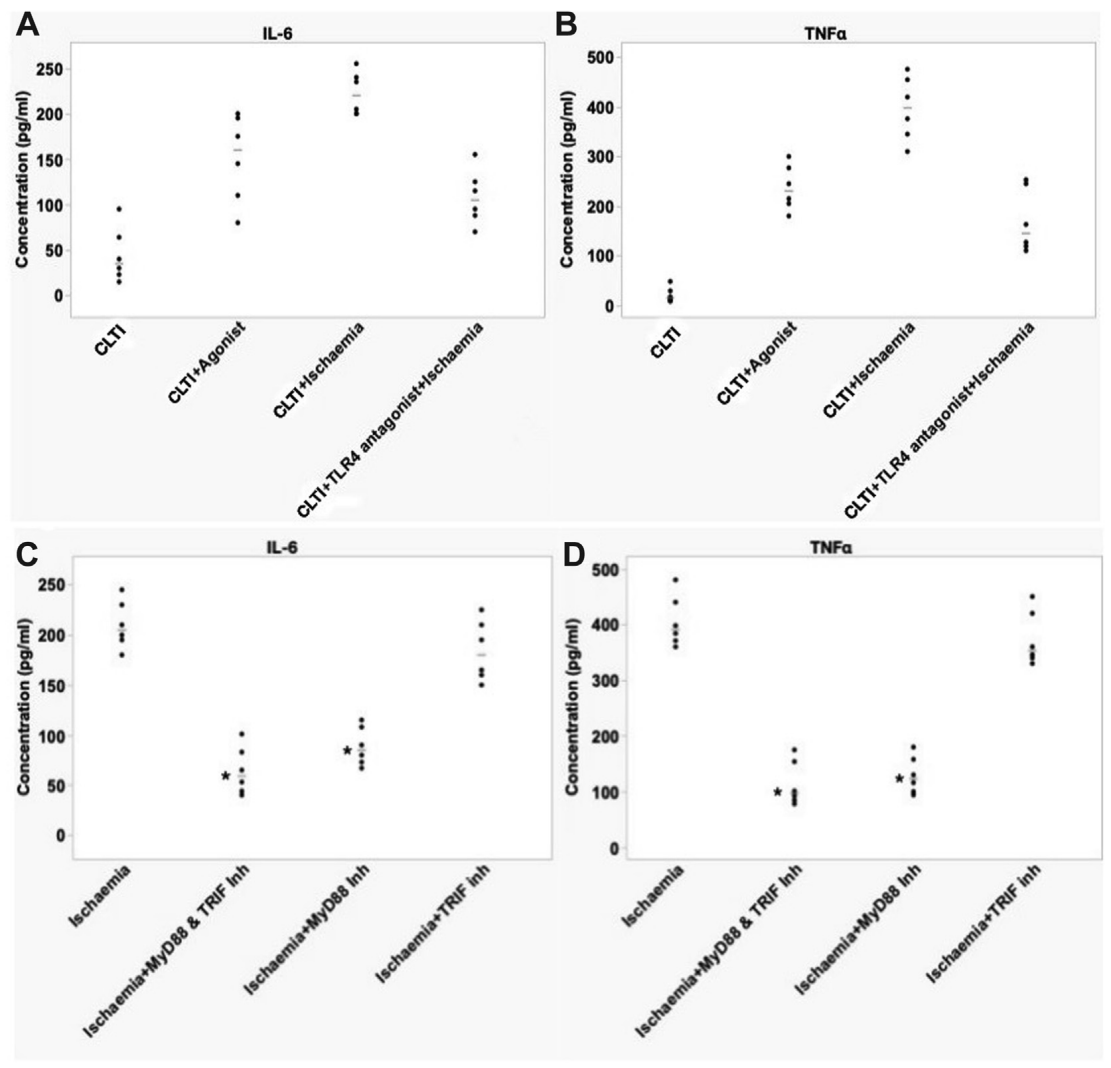
Effect of Toll-like receptor 4 (TLR4) antagonism and ischemia on myotubes from patients with chronic limb-threatening ischemia (CLTI). (**A**) TLR4 antagonist pretreatment attenuates ischemia-induced IL6 release (n = 6; *P* < .05; Mann-Whitney *U* test). (**B**) TLR4 antagonist pretreatment attenuates ischemia-induced tumor necrosis factor α (*TNF-*α) release (n = 6; *P* < .05; Mann-Whitney *U* test). Enzyme-linked immunosorbent assay analyses of (**C**) interleukin 6 (*IL-6*) and (**D**) *TNF-α* from cultured myotubes from patients with CLTI, with or without pretreatment with MyD88 and TRIF inhibitor (n = 6). ∗*P* < .05; Mann-Whitney *U* test.

Western blot analyses of cell lysates from patients with CLTI confirmed the presence of the adaptor proteins MyD88 and TRIF and their expressions were independent of exposure to simulated ischemia in culture (Fig 2). Pretreatment of myotubes from patients with CLTI with MyD88 inhibitor before simulated ischemia resulted in decreased IL6 (median, 5pg/mL 9; range, 40-10 pg/mL 1 vs median, 205 pg/mL; range, 180-245 pg/mL) and TNF-α (median, 97pg/mL; range, 78-175 pg/mL vs median, 391 pg/mL; range, 360-480 pg/mL) release compared with myotubes with no pretreatment before simulated ischemia (Fig 3) and down-regulation of cleaved caspase-3 (median, 56; range, 42-78 vs median, 128; range, 102-160; *P* < .05; Mann-Whitney *U* test) (Fig 3). However, myotubes pretreated with TRIF inhibitor disclosed no significant changes in IL6 and TNF-α release or cleaved caspase-3 expression (*P* > .05; Mann-Whitney *U* test). Further, the inhibition of the adaptor proteins before simulated ischemia was associated with reduced expression of HSP60 (median, 1600; range, 1000-2100 vs median, 3800; range, 3000-4200) and HSP70 (median, 3800; range, 2700-5000 vs median, 7000; range, 5400-7800) (*P* < .05; Mann-Whitney *U* test).

### In vivo experiments

**Fig 4:**
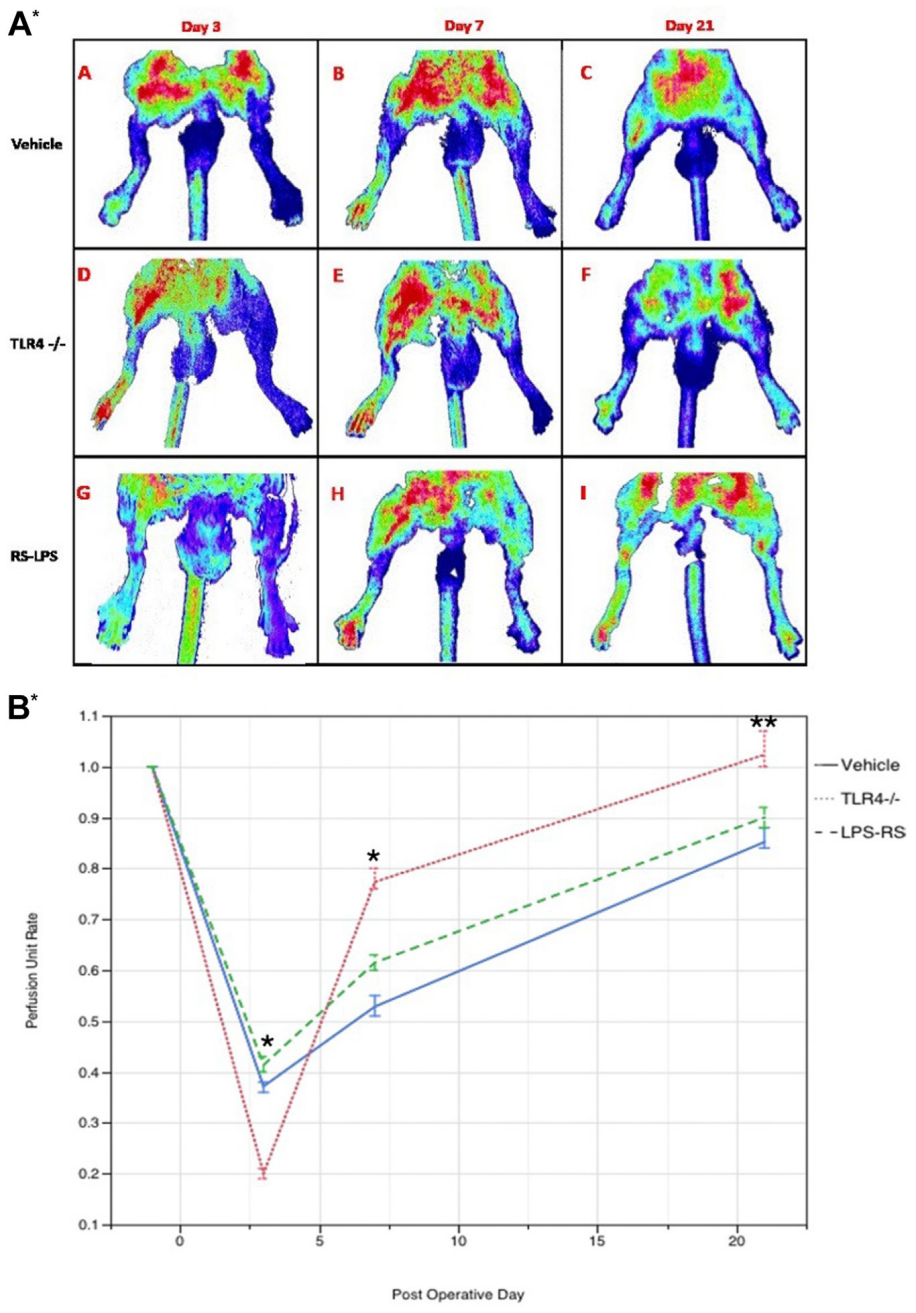
(**A**) Representative laser Doppler perfusion images obtained with mice positioned supine under the scan head on a low-temperature heating pad. Serial color-coded perfusion images (**A-C**) of 12-week-old C57BL/6 mice on postoperative days 3, 7, and 21, respectively. (**D**-**F**) Twelve-week-old C57BL/6 mice with genetically deleted Toll-like receptor 4 (*TLR4*) on postoperative days 3, 7, and 21, respectively. (**G-I**) Twelve-week-old C57BL/6 treated with exogenous TLR4 inhibition on postoperative days 3, 7, and 21, respectively. (**B**) Perfusion recovery after surgery. The ischemic/nonischemic lower limb perfusion ratio showed a significant difference on days 3, 7, and 21 between the TLR4^−/−^ group and control and TLR4 antagonist (LPS-RS) groups (∗*P* < .001 at days 3 and 7; ∗∗*P* = .01 at day 21; Kruskal-Wallis test). The difference between each group was also significant (*P* < .005; Mann-Whitney *U* test) on days 3, 7, and 21. *RS-LPS*, lipopolysaccharide from *Rhodobacter sphaeroides*.

Analysis of the laser Doppler images showed a significant reduction in perfusion in the operated hindlimb at day 3 after surgery in all groups (perfusion unit: control [n = 6], median, 0.38; TLR4^−/−^ [n = 6], median, 0.2; LPS-RS [n = 6], median, 0.41), with the decrease being more significant in the TLR4^−/−^ group when compared with control and LPS-RS groups at this time point (*P* < .05; Mann-Whitney *U* test) (Fig 4). Perfusion in the sham-operated groups showed no significant differences between the groups at all time points. Slower blood flow recovery was observed in the control group in comparison to TLR4^−/−^ and LPS-RS groups at day 3 (perfusion unit: control [n = 6], median, 0.38; TLR4^−/−^ [n = 6], median, 0.2; LPS-RS [n = 6], median, 0.41), day 7 (perfusion unit: control [n = 6], median, 0.51; TLR4^−/−^ [n = 6], median, 0.79; LPS-RS [n = 6], median, 0.61), and day 21 (perfusion unit: control [n = 6], median, 0.84; TLR4^−/−^ [n = 6], median, 1.1; LPS-RS [n = 6], median, 0.90) (*P* < .05; Kruskal-Wallis test) (Fig 4).

**Fig 5:**
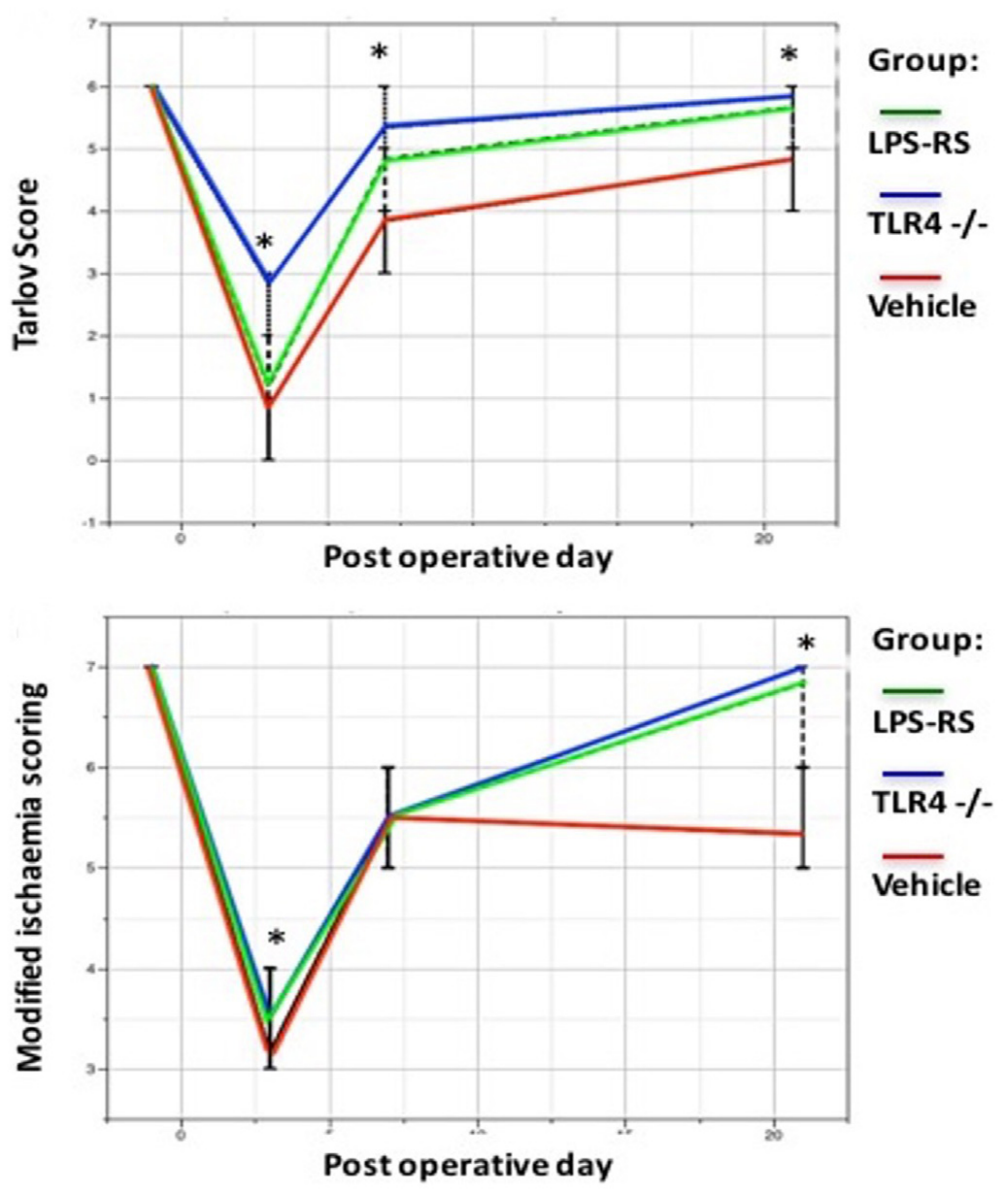
Functional and ischemic scoring of the mice in all groups showed faster recovery in Toll-like receptor (TLR4)^*−/−*^ and lipopolysaccharide (LPS-RS) groups compared with the vehicle group (∗*P* < .05 between groups; Kruskal-Wallis test; n = 6).

All mice showed low functional performance after surgery with gradual improvement by day 21. Mice in TLR4^−/−^ and LPS-RS groups restored their functional status to near baseline by day 21, whereas the control group had lower performance even at day 21 (*P* < .05; Kruskal-Wallis test) (Fig 5). The blood gas analyses of blood samples taken via cardiac puncture at the time of sacrificing the animals showed no significant differences between the ischemic and sham-operated mice in either the control or TLR4^−/−^ groups when the following parameters were quantified: pH, pO_2_, pCO_2_, hemoglobin, lactate, Na, K, and SaO_2_. The random blood glucose levels in the ischemic and sham-operated TLR4^−/−^ groups were significantly higher compared with ischemic and sham-operated control groups at days −1, 3, 7, and 21 (*P* < .05; Kruskal-Wallis test). The ELISA quantification of IL-6 showed significantly increased levels in the control group compared with TLR4^−/−^ and LPS-RS groups at days 3, 7, and 21 after surgery (*P* < .05; Kruskal-Wallis test). Similarly, ELISA quantification of TNF-α showed significantly increased levels in the control group compared with TLR4^−/−^ and LPS-RS groups at days 3, 7, and 21 after surgery (*P* < .05; Kruskal-Wallis test).

**Fig 6:**
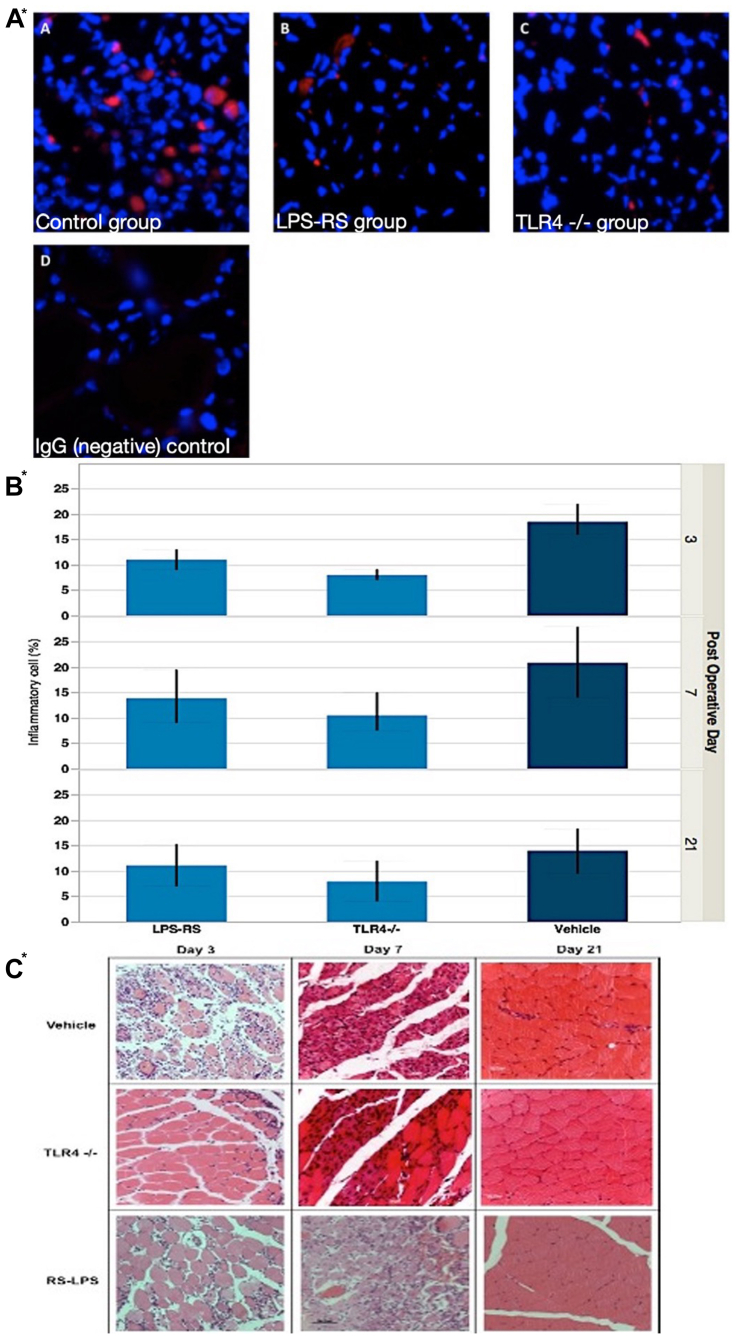
(**A**) Fluorescent immunohistochemical staining for cleaved caspase-3 (red color) in C57BL/6 mouse gastrocnemius muscle, post hindlimb ischemia day 21 (paraffin embedded, DAPI [blue] and AlexaFluor 647 [red] stains, 3-micron-thick section; original magnification ×40). Representative images from (**A**) control group, (**B**) lipopolysaccharide (LPS-RS) group, (**C**) Toll-like receptor 4 (*TLR4*)^−/−^ group, and (**D**) IgG (negative) control. (**B**) Inflammatory cell quantification in hematoxylin and eosin (H&E)-stained ischemic gastrocnemius muscle showed less inflammatory cell infiltration in both TLR4^−/−^ and LPS-RS groups when compared with the control group at each time point (*P* < .05; Kruskal-Wallis test; n = 6). (**C**) Representative H&E staining of ischemic gastrocnemius muscle on postoperative days 3, 7, and 21 (scale bar, 50 μm).

Fluorescent immunohistochemical staining for cleaved-caspase 3 showed less expression of this protein in TLR4-inhibited groups at day 21 (Fig 6). Inflammatory cell quantification, carried out using ImageJ 1.4s (US National Institutes of Health) in hematoxylin and eosin-stained, paraffin-embedded ischemic gastrocnemius muscle sections after surgery showed less inflammatory cell infiltration in both TLR4^−/−^ and LPS-RS groups when compared with the control group at each time point (*P* < .05; Kruskal-Wallis test) (Fig 6.) The median percentages of inflammatory cell infiltration in the control, TLR4^−/−^, and LPS-RS groups were calculated as 19.0%, 7.0%, and 10.5%, respectively, at day 3; 21.0%, 10.0%, and 14.0%, respectively, at day 7; and 14.0%, 8.0%, and 10.5%, respectively, at day 21. TLR4^−/−^ mice and mice given LPS-RS demonstrated attenuated histological evidence of ischemia-induced inflammation after hindlimb ischemia as compared with control mice at days 3, 7, and 21. The hematoxylin and eosin examination of the hindlimb skeletal muscles in sham groups showed no obvious histological differences between the control, TLR4^−/−^, and LPS-RS groups at days 3, 7, and 21.

## Discussion

Understanding the pathophysiology of the ischemia-induced inflammatory damage in CLTI may provide a potential missing link in improving functional outcomes along with successful revascularization. It is thought that the muscle dysfunction in CLTI is potentially due to ischemia-induced muscle damage, in which inflammation plays a central role.^5^^,^^12^

We showed that TLR4 is expressed in both ischemic and nonischemic human skeletal muscle, with increased expression in the ischemic samples. TLR4-mediated NF-κB and JNK pathways have been shown to be involved in myocardium ischaemia.^13^^,^^14^ Here, P-NF-κB and pJNK, activated forms of NF-κB and JNK, were significantly upregulated in the ischemic samples. The high expression of these transcription proteins suggests that TLR4 is not only upregulated, but also activated. Further, the increase in cleaved caspase-3, a marker for apoptosis, suggests that significant tissue damage occurs even within clinically viable lower limb muscle. This finding supports previous studies implicating TLR-induced apoptotic cell death in the pathophysiology of ischemia-induced cell damage.^15^ Immunofluorescence staining of the muscle biopsies revealed the presence of TLR4 on neutrophils, endothelium, and macrophages.

In vitro experiments showed that exposure of mature myotubes to nutrition depletion, hypoxia, and hypercapnia resulted in a significant time-dependent increase in the percentage of apoptotic cells and inflammatory cytokine release, which are features of skeletal muscle ischemia in vivo. Simulated ischemia in cultured human myotubes also led to the upregulation of TLR4 and activation of its downstream signaling pathway with increased cytokine release and apoptosis in the ischemic human myotubes. Further, inhibition of TLR4 before ischemia was associated with inhibition of the signaling pathway and reduced ischemia-induced apoptosis. Moreover, amplified cytokine release and increased expression of HSP60 and HSP70 were demonstrated in ischemic skeletal muscle in vitro. Inhibition of the MyD88-dependent signaling pathway attenuated inflammatory cytokine production in the presence of ischemia-induced TLR4 activation. These data are in keeping with previous findings of accelerated muscle regeneration in hindlimb ischemia in MyD88 knockout mice,^16^ and supports the involvement of MyD88 in the inflammatory response to ischemia, which may be a critical step in the development of skeletal muscle damage. Furthermore, TLR4 antagonism was associated with reduced inflammatory cytokine release and downregulation of HSP60 and HSP70 expressions. This finding suggests a potential pathway where TLR4 and its endogenous ligands contribute to a positive feedback loop to maintain a proinflammatory environment during ischemia. The in vivo experiments showed that TLR4 is upregulated in ischemic skeletal muscle. TLR4^−/−^ mice and mice given LPS-RS, a TLR4 antagonist, exhibited decreased systemic IL6 and TNF-α levels after hindlimb ischemia, implicating the role of TLR4 in ischemia-induced systemic inflammatory cytokine production. Both endogenous and exogenous inhibition of TLR4 were associated with decreased inflammatory cell infiltration and diminished apoptosis in the ischemic limb as compared with the control group.

The overall limitation of our study is the need for a more in-depth analysis of the signaling pathways and mechanisms involved, because the crosstalk between the different signaling pathways can affect the final response. Further studies are required to explore different drug delivery routes and timing and the dose-response effect of TLR4 antagonists. Although TLR antagonists have been shown clearly to be protective by directly inhibiting inflammatory gene transcription and decreasing the secretion of inflammatory cytokines in several conditions, preconditioning with low-dose TLR agonists may also be an effective strategy to protect tissue against subsequent detrimental insults such as reperfusion injury. This result highlights the importance of administering specific TLR agonists and antagonists during the relevant phase of the pathological process to achieve an optimal balance of TLR activation and inhibition.^17^ This study also has specific limitations regarding the in vivo model: a chronic model of ischemia with gradual occlusion was not performed in our experiments, mainly because we aimed to develop a cost-effective model that can be used in most laboratories; we did not evaluate older mice, which may demonstrate more similar responses to our CLTI patients, again owing to practical husbandry and cost considerations.

The TLR4-induced inflammatory response has been shown to encourage angiogenesis and collateral artery formation in various ischemia/reperfusion models, suggesting the dual role of TLR4 in inflammation and angiogenesis. However, it has been reasoned that the fine-tuning of TLR4 and its associated signaling pathway can eliminate the undesired effect of TLR4 inhibition on collateral artery formation.^18^ van den Borne et al^19^ studied the effect of systemic inhibition of TLR4 on perfusion recovery in a mouse model for angiogenesis. They found that inhibition of TLR4 by its specific inhibitor TAK-242 in a mouse model of hindlimb ischemia did not negatively influence perfusion recovery after ischemia, despite its potential inhibitory effects on angiogenesis.^19^ Moreover, TLR4 can interplay with other inflammatory signaling pathways to balance the final response to ischemia. Inflammation is involved in both muscle regeneration and angiogenesis; however, a regulated balance is required to prevent unnecessary inflammation-induced tissue damage, while preserving the positive effects. It has been reported that unopposed TLR4 activation in skeletal muscle ischemia slows regeneration and also angiogenesis, whereas TLR2, which is simultaneously upregulated by TLR4, plays a protective role in modifying the TLR4 effect.^20^ Additionally, the other components of the adaptive immune system such as T-cell-mediated responses are further potential areas of future research to explore the possible interactions with the TLR4 signaling pathway. The inhibition of TLR4 can also be considered in adjunct with other novel strategies. Decreasing the ischemia-induced inflammatory response may improve the environment/niche to enable growth factors or stem cells to function better.

The potential therapeutic role of TLR4 antagonists should be considered as adjuvant therapy in PAD. Muscle changes including inflammation are documented in patients with intermittent claudication. We envisage a potential therapeutic role of TLR4 antagonists as an adjuvant therapy where earlier intervention before severe muscle damage, alongside revascularization may offer more benefit than treating patients with late-stage CLTI. The risk-benefit ratio would need to be determined based on any side-effects of treatment at each stage.

**Fig 7:**
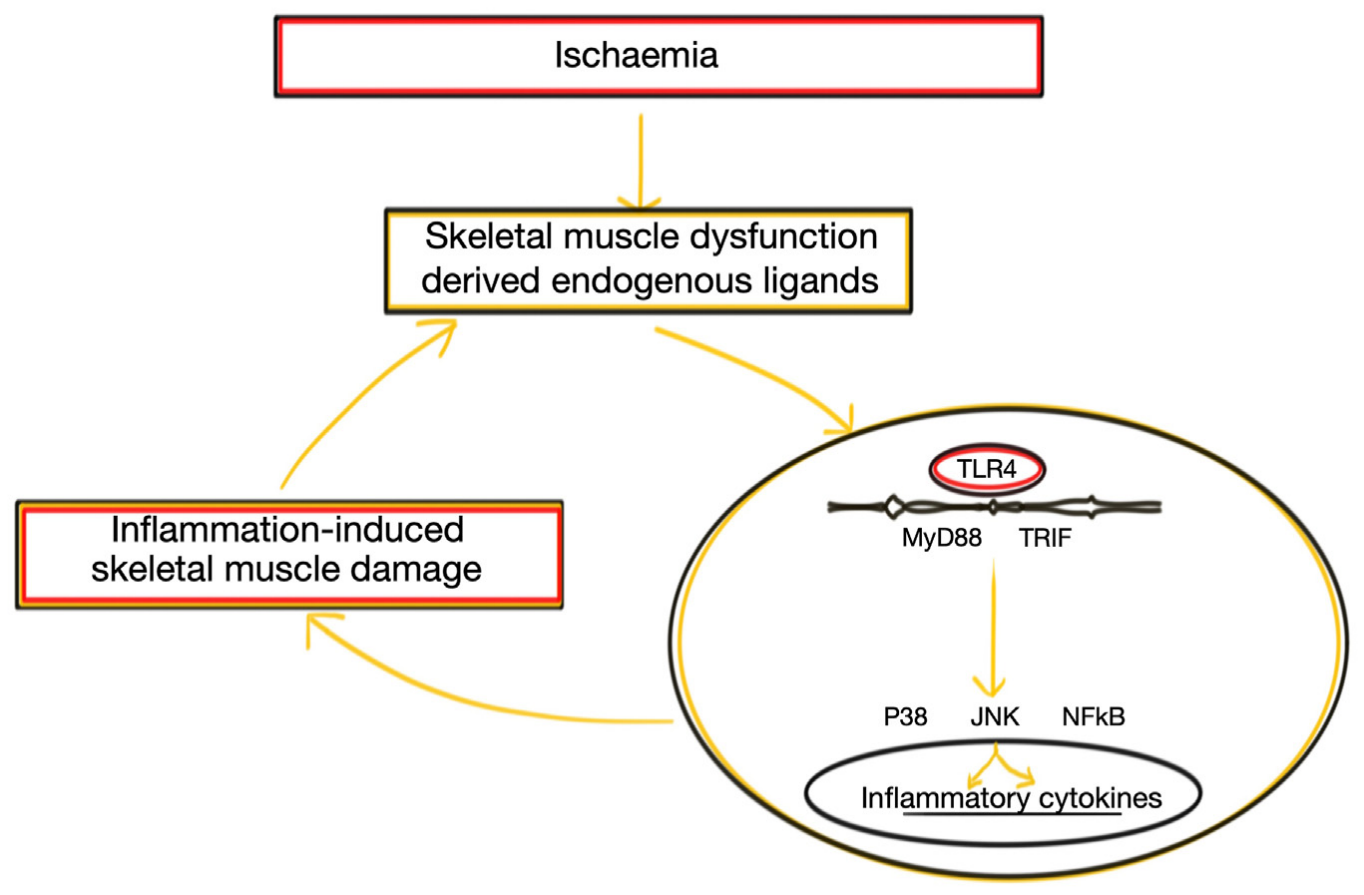
Schematic showing the potential link between ischemia-induced Toll-like receptor 4 (*TLR4*) activation and the resulting tissue damage. Ischemia leads to muscle damage with the release of inflammatory cytokines. Upon activation of TLR4, adaptor proteins (MyD88 and TRIF) and transcription proteins (P38, JNK, and nuclear factor κB [NF-κB]) are recruited to activate the inflammatory gene transcription.

In summary, this study investigated the role of TLR4-mediated inflammatory responses in ischemic skeletal muscle. TLR4 was found to be upregulated and activated in ischemic skeletal muscle in patients with CLTI, and TLR4 signaling contributed to ischemia-induced inflammation and cell/tissue damage in vitro and in vivo (Fig 7). Modulating TLR4 signaling in vivo was associated with attenuation of skeletal muscle damage. This work highlights the potential therapeutic role of TLR4 inhibition in patients with CLTI. It is conceivable that pharmacological targeting of TLR4 in patients with CLTI together with improving hemodynamics may improve both functional and clinical outcomes.

## Author Contributions

Conception and design: AN, XS, DB, DA, JT

Analysis and interpretation: AN, XS, JT

Data collection: AN, HP

Writing the article: AN, JT

Critical revision of the article: AN, XS, DB, DA, JT

Final approval of the article: AN, HP, XS, DB, DA, JT

Statistical analysis: AN, JT

Obtained funding: JT, DA

Overall responsibility: JT

## Disclosures

None.

## References

[1] Conte M.S., Bradbury A.W., Kolh P. (2019). Global vascular guidelines on the management of chronic limb-threatening ischemia. Eur J Vasc Endovasc Surg.

[2] Almasri J., Adusumalli J., Asi N. (2019). A systematic review and meta-analysis of revascularization outcomes of infrainguinal chronic limb-threatening ischemia. Eur J Vasc Endovasc Surg.

[3] Kumar B.N., Gambhir R.P. (2011). Critical limb ischemia-need to look beyond limb salvage. Ann Vasc Surg.

[4] Pipinos, Judge A.R., Selsby J.T. (2007). The myopathy of peripheral arterial occlusive disease: part 1. Functional and histomorphological changes and evidence for mitochondrial dysfunction. Vasc Endovasc Surg.

[5] Pipinos, Judge A.R., Selsby J.T. (2008). The myopathy of peripheral arterial occlusive disease: Part 2. Oxidative stress, neuropathy, and shift in muscle fiber type. Vasc Endovasc Surg.

[6] Rigato M., Monami M., Fadini G.P. (2017). Autologous cell therapy for peripheral arterial disease: systematic review and meta-analysis of Randomized, nonrandomized, and noncontrolled studies. Circ Res.

[7] Drexler S.K., Foxwell B.M. (2010). The role of toll-like receptors in chronic inflammation. Int J Biochem Cell Biol.

[8] Piccinini A.M., Midwood K.S. (2010). DAMPening inflammation by modulating TLR signalling. Mediators Inflamm.

[9] Seneviratne A.N., Sivagurunathan B., Monaco C. (2012). Toll-like receptors and macrophage activation in atherosclerosis. Clin Chim Acta.

[10] Shimamoto A., Chong A.J., Yada M. (2006). Inhibition of Toll-like receptor 4 with eritoran attenuates myocardial ischemia-reperfusion injury. Circulation.

[11] Corbu A., Scaramozza A., Badiali-DeGiorgi L. (2010). Satellite cell characterization from aging human muscle. Neurol Res.

[12] Pipinos I.I., Swanson S.A., Zhu Z. (2008). Chronically ischemic mouse skeletal muscle exhibits myopathy in association with mitochondrial dysfunction and oxidative damage. Am J Physiol Regul Integr Comp Physiol.

[13] Chong A.J., Shimamoto A., Hampton C.R. (2004). Toll-like receptor 4 mediates ischemia/reperfusion injury of the heart. J Thorac Cardiovasc Surg.

[14] Takeda K., Akira S. (2004). TLR signaling pathways. Semin Immunol.

[15] Patel H., Shaw S.G., Shi-Wen X., Abraham D., Baker D.M., Tsui J.C. (2012). Toll-like receptors in ischaemia and its potential role in the pathophysiology of muscle damage in critical limb ischaemia. Cardiol Res Pract.

[16] Sachdev U., Cui X., McEnaney R., Wang T., Benabou K., Tzeng E. (2012). TLR2 and TLR4 mediate differential responses to limb ischemia through MyD88-dependent and independent pathways. PLoS One.

[17] Navi A., Patel H., Shaw S., Baker D., Tsui J. (2013). Therapeutic role of toll-like receptor modification in cardiovascular dysfunction. Vascul Pharmacol.

[18] Murad S. (2014). Toll-like receptor 4 in inflammation and angiogenesis: a double-edged sword. Front Immunol.

[19] van den Borne P., Bastiaansen A.J., de Vries M.R., Quax P.H., Hoefer I.E., Pasterkamp G. (2014). Toll-like receptor 4 inhibitor TAK-242 treatment does not influence perfusion recovery in tissue ischemia. J Cardiovasc Pharmacol.

[20] Xu J., Benabou K., Cui X. (2015). TLR4 deters perfusion recovery and upregulates toll-like receptor 2 (TLR2) in ischemic skeletal muscle and endothelial cells. Mol Med.

