# Supplementary material for: Role of toll-like receptor 4 in skeletal muscle damage in chronic limb-threatening ischemia
Source: JVS Vasc Sci. 2024 Feb 11;5:100194. doi: 10.1016/j.jvssci.2024.100194 (PMC10951510; doi:10.1016/j.jvssci.2024.100194)
Supplement: Online Supplement [file mmc1.docx]

**Online Supplement for METHODS:**

**Human tissue collection**

Written informed consent was obtained from patients with prior approval from the Royal Free & Medical School Research Ethics Committee (REC Ref: 29-2000). Human skeletal muscle biopsies were taken from the medial head of gastrocnemius from patients undergoing major lower limb amputation for CLTI (n=6) and from patients with no PAD undergoing saphenous vein harvesting for CABG surgery with ankle-pressure index >0.9 (n=6). All biopsies were taken from areas with no tissue necrosis or active infection. Further, to allow blind analysis, all samples were labelled with serial numbers prior to storage. The tissue was snap frozen in liquid nitrogen and stored at -80°C until use.

**Western blotting**

Denatured protein samples were loaded onto 10% Tris-Glycin gels, placed into XCell Sure Lock electrophoresis device and run at 125V for 1.5-2 hours. The proteins were transferred onto nitrocellulose membrane (Hybond-C extra; GE Healthcare Life Sciences, Buckinghamshire, UK) at 30V for 90 minutes. The membranes were blocked with 5% milk solution, followed by incubation with the primary antibodies: TRIF (ab13810, Abcam, 1:1000), TLR4 (ab22.48, Abcam, 1:500), HSP70 (sc-373867, Anta Cruz, 1:1000), HSP60 (sc-136291, Santa Cruz, 1:1000), MyD88 (ab2064, Abcam, 1:1000), P-P38 (D3F9, Cell Signalling, 1:1000), P38 (9212, Cell Signalling, 1:1000), JNK (9252, Cell Signalling, 1:1000), P-JNK (9251, Cell Signalling, 1:1000), NFKB (C22B4, Cell Signalling, 1:1000), P-NFKB (93H1, Cell Signalling, 1:2000), Cleaved Caspase-3 (9661, Cell Signalling, 1:1000), β tubulin loading (ab4074, Abcam, 1:1000). The membranes were developed using chemiluminescent substrate (Amersham ECL Plus™ Western Blotting Detection Reagent; GE Healthcare Life Sciences) and the blots were developed against photographic film (Amersham Hyperfilm ECL, GE Healthcare Life Sciences). Band intensities were determined by densitometry using computer software ImageJ 1.45s (US National Institutes of Health, Bethesda, Maryland, USA).

**Immunohistochemistry & Immunocytochemistry**

Human and mouse skeletal muscle biopsies were fixed in 10% formalin and embedded in paraffin, then cut by microtome to approximate 10-micron thickness and affixed onto slides. Primary antibodies used were as follow: TLR4 (mouse, ab22048, Abcam, 1:100), CD34 (mouse, ab9088, Abcam, 1:100), CD68 (mouse, M0814, DakoCytomation, 1:100), CD31 (mouse, M0823, DakoCytomation, 1:20). Secondary antibodies used were: Alexa Fluor® 488 Anti-Rabbit IgG (gout, A-11008, Life Technologies, 1:200), Alexa Fluor™ 594 Anti-Rabbit IgG (gout, A-11012, Life Technologies, 1:200), Alexa Fluor® 594 Anti-Mouse IgG (chicken, A-11012, Life Technologies, 1:200), Alexa Fluor® 488 Anti-Mouse IgG (chicken, A-1101, Life Technologies, 1:200), Alexa Fluor® 488 Anti-Rabbit IgG (gout, A-11008, Life Technologies, 1:200). Cultured human myotubes were fixed on 8 chamber culture slides to visualize the presence of Desmin, marker for human skeletal muscle cell. Coverslips were mounted using VECTASHIELD® containing DAPI. Images were viewed using Axiotop 2 Mot plus microscope with Axiovision software and MRm camera (Carl Zeiss, Gottingen, Germany).

**Haematoxylin & Eosin (H&E) staining**

Slides were deparaffinised and rehydrated, then stained in Harris’s Haematoxylin (Surgipath Europe Ltd, Peterborough, UK). The slides were decolourised and further treatment with Lithium Carbonate for 3 seconds was performed, before washing in tap water and counterstaining in 1% Eosin for 15 seconds. Dehydration in 100% alcohol and xylene was carried out, before mounting sections with cover slips using DPX resin (BDH Guir^®^). Slides were examined under a light microscope and pictures taken with Zeiss Axiotop 2 Mot. Inflammatory cell quantification was carried out using ImageJ 1.4s (US National Institutes of Health) on H&E-stained paraffin-embedded left hindlimb skeletal muscle sections obtained from *in vivo* experiments.

**Human skeletal muscle cell culture**

Human myoblasts were isolated, cultured to myotubes and then exposed to simulated ischaemia. The isolated myoblasts were examined every 48 hours to monitor for 80% confluence before induction to differentiate into myotubes. Same numbers of cultured cells were placed in culture plates to normalise the quantity of proteins in all the experiments.

Human myoblasts were cultured in 50ml tubes containing 20ml in Dulbecco’s modified Eagle’s medium (DMEM, Gibco, Paisley, Scotland) supplemented with 20% foetal calf serum, penicillin (100 U/ml), amphotericin B (25 µg/ml), streptomycin (100 µg/ml, all from Gibco).

To culture the myoblasts to myotubes, the myoblasts were plated onto 6 well culture plates (BD Falcon™, cat. no. 351143) or 8-well chamber slides (Falcon) and cultured in Skeletal Muscle Cell Growth Medium (SMM, Foetal Calf Serum, Fetuin, Epidermal Growth Factor, Basic Fibroblast Growth Factor, Insulin and Dexamethasone; 23060; PromoCell, Germany) supplemented with 20% foetal calf serum, penicillin (100 U/ml), amphotericin B (25 µg/ml), streptomycin (100 µg/ml). At 80% confluence, cells were exposed to differentiation medium containing DMEM with 2% horse serum, penicillin (100U/ml), streptomycin (100 µg/ml) and amphotericin B (25 µg/ml) (all from Gibco). In case of any contamination with endothelial cells, the culture medium was changed to a mixture of 20% DMEM and 20% SMM (50:50) for 48 hours, and then 20% DMEM only. The culture medium was switched back to 20% SMM when no endothelial cells could be visualized under the microscope. To investigate the functional consequences of ischaemia in cultured human myotubes, a model of simulated ischaemia was used (1). Briefly, myotubes were exposed to 20% CO_2_+80% N_2_ (British Oxygen, Luton, UK) in hypoxic chambers (Modular Incubator Chamber, MIC-101; Billups-Rothenberg, Del Mar, CA, USA). The chamber was flushed with gas at 10 l/min for 20 minutes and then sealed. The apparatus was placed in an incubator at 37°C for 8 and 72h. Control myotubes were maintained under normoxic conditions (21% O_2_+5% CO_2_) for 8h.

**Protein inhibitors and TLR4 antagonism**

To investigate the TLR4 related downstream signalling pathway in simulated ischaemia, protein inhibitors were used to manipulate the signalling pathway at the level of both adaptor and transcription proteins *in vitro*. The following inhibitors were added 6 hours prior to exposure to simulated ischaemia to inhibit the adaptor proteins: MyD88 inhibitor (20μM; tlrl-pimyd; Invivogen) and TRIF inhibitor (20μM; tlrl-pitrif; Invivogen). The following inhibitors were used to inhibit the transcription proteins: NF-kB inhibitor (10μM; Celastrol ant-cls; Invivogen) was added 6 hours prior to exposure to simulated ischaemia; P38 inhibitor (10μM; SB203580; SelleckChem, Houston, USA) was added 4 hours prior to exposure to simulated ischaemia; JNK inhibitor (20μM; 420119; Merck Chemicals Ltd, Nottingham, UK) was added 4 hours prior to exposure to simulated ischaemia. The following inhibitors were used to inhibit the TLR4 receptor: Anti-human TLR4 neutralizing antibody (10 μg/ml; mabg-htlr4; Invivogen) was added 2 hours prior to exposure to simulated ischaemia and TLR4 antagonist Lipopolysaccharide from *Rhodobacter sphaeroides* (RS-LPS), LPS-RS Ultrapure (10 μg/ml; tlrl-prslps; Invivogen) was added 4 hours prior to exposure to simulated ischaemia. The RS-LPS was also used for the *in vivo* experiments to inhibit TLR4 prior to the hindlimb ischaemia.

**ELISA**

The sandwich ELISA technique was carried out to detect the presence and to quantify the concentration of inflammatory cytokines (IL6, TNFα and INFα) and endogenous ligands (HSP60 and HSP70) in the *in vitro* experiments and inflammatory cytokines (IL6 and TNFα) in the *in vivo* experiments. The following ELISA kits were used: Human VeriKine^TM^ INFα (41100; PBL Assay Science, USA), Human total HSP60 (1800-2; R&D Systems, USA), Human IL6 (D6050; R&D Systems), Human TNFα (DTA00C; R&D Systems), Human / Mouse total HSP70 (DYC1663-2; R&D Systems), Mouse IL6 (M6000B; R&D Systems) and Mouse TNFα (MTA00B; R&D Systems).

**Mouse model of hindlimb ischaemia**

All procedures were performed in accordance with Home Office legislation, under Project Licence number 70/7087. ARRIVE guidelines were considered and followed to improve the design, analysis and reporting of the *in vivo* experiments (2). Male C57BL/6 mice of 12 weeks of age were kept pathogen-free in groups of 6 in a strict 12-hour night and day cycle. They were fed standard chow with free access to water. The three groups of mice were: A) Control group (wild type; n=18); B) RS-LPS group (wild type – TLR4 antagonist; n=18); C) TLR4 -/- group (n=18). In addition, nine animals per group were sham operated. Animals were anesthetized by intraperitoneal injection of 2% Avertin, 2, 2, 2-Tribromoethanol, (0.25 ml/g). To maintain the anaesthesia, 1% isoflurane mixed in 100% oxygen with flow of 0.8 l/min was used during the procedure. All the operations were performed over two consequent days by same operator. After the mouse was anaesthetized, hair on the abdominal wall, pelvis and the upper legs was shaved using an electronic shaver. The skin was cleaned using alcohol spray. A skin incision was made on the upper thigh of the mouse. The inguinal ligament and the upper half of the femoral artery were exposed using sharp and blunt dissection. The femoral artery was ligated with sterile 7-0 Prolene suture at the level of inguinal ligament proximally and just above the popliteal artery distally. All the side branches were ligated with 7-0 Prolene suture. The skin incision was closed with 5-0 silk suture. Animals were nursed for a period of one-hour post-surgery in a recovery area on a 37^°^C warming pad, with buprenorphine (0.1 mg/kg, SC) to manage the pain. The overall well-being of animals was checked daily with specific assessment of function performed on post-surgical day 3, 7 and 21. Tissue necrosis, limping and muscle flexion were recorded using the Tarlov and Modified Ischaemia scoring systems to assess post-operative function (3). Animals were sacrificed (n=6 per ischaemic groups and n=3 per sham groups at each time point) and hindlimb tissue and serum samples were collected at day 3, 7 and 21.

**Haemodynamics and Laser Doppler Imaging**

To monitor tissue perfusion *in vivo*, anaesthetized mice were positioned under the scanner head (Moor FLPI; Moor Instruments Ltd, Devon, UK) on a low temperature heating pad to maintain normal body temperature of 37°C, with 30cm distance between the animal and the scan head. The mice were placed in supine position and serial color-coded perfusion images were taken at days 1, 3 and 21 post-operation. The images were then analysed using Moor FLPI review V3.0 software. The ischaemic hindlimb perfusion was described as a ratio of the color-coded perfusion image of the ischaemic limb to the non-ischaemic one.

**Power calculations and statistical analysis**

For *in vivo* studies, 6 animals of each group were used for each time point. This number was chosen based on published studies of TLR changes in ischaemia as well as studies employing similar analyses in mouse hindlimb ischaemia models (4, 5). JMP® 11.0.0 software (SAS institute, NC, USA) was used to present, describe and analyse the data from this study. The Chi-square test was used to compare the demographics between groups. Data from human tissue experiments were described as medians and ranges and compared with the Mann Whitney U test. Data from *in vitro* experiments were described as medians and ranges and compared with the Kruskal Wallis test. Data from *in vivo* experiments were described as medians and ranges and compared with the ANOVA test.

References:

1. Joshi D, Patel H, Baker DM, Shiwen X, Abraham DJ, Tsui JC. Development of an in vitro model of myotube ischemia. Laboratory investigation; a journal of technical methods and pathology. 2011;91(8):1241-52.

2. Kilkenny C, Browne WJ, Cuthi I, Emerson M, Altman DG. Improving bioscience research reporting: the ARRIVE guidelines for reporting animal research. Vet Clin Pathol. 2012;41(1):27-31.

3. Brenes R, Jadlowiec C, Bear M, Hashim P, Protack C, Li X, et al.Toward A Mouse Model of Hind Limb Ischemia to Test Therapeutic Angiogenesis. J Vasc Surg. 2012;56(6): 1669–1679.

4. Shimamoto A, Chong AJ, Yada M, Shomura S, Takayama H, Fleisig AJ, et al. Inhibition of Toll-like receptor 4 with eritoran attenuates myocardial ischemia-reperfusion injury. Circulation. 2006;114(1 Suppl):I270-4.

5. Corbu A, Scaramozza A, Badiali-DeGiorgi L, Tarantino L, Papa V, Rinaldi R, et al. Satellite cell characterization from aging human muscle. Neurological research. 2010;32(1):63-72.
